# Calcineurin inhibitor Tacrolimus impairs host immune response against urinary tract infection

**DOI:** 10.1038/s41598-018-37482-x

**Published:** 2019-01-14

**Authors:** Diba Emal, Elena Rampanelli, Nike Claessen, Frederike J. Bemelman, Jaklien C. Leemans, Sandrine Florquin, Mark C. Dessing

**Affiliations:** 1 Department of Pathology, Academic Medical Center, University of Amsterdam, Amsterdam, The Netherlands; 2 Renal Transplant Unit, Department of Internal Medicine, Academic Medical Center, University of Amsterdam, Amsterdam, The Netherlands

## Abstract

Calcineurin inhibitor Tacrolimus, is a potent immunosuppressive drug widely used in order to prevent acute graft rejection. Urinary tract infection (UTI) is the most frequent infectious complication in renal transplant patients and long-term use of Tacrolimus might be involved in higher susceptibility to bacterial infections. It remains largely unknown how Tacrolimus affects the host innate immune response against lower and upper UTI. To address this issue, we used experimental UTI model by intravesical inoculation of uropathogenic *E*.*coli* in female wild-type mice pre-treated with Tacrolimus or solvent (CTR). We found that Tacrolimus pre-treated mice displayed higher bacterial loads (cystitis, pyelonephritis and bacteremia) than CTR mice. Granulocytes from Tacrolimus pre-treated mice phagocytized less *E*. *coli*, released less MPO and expressed decreased levels of CXCR2 receptor upon infection. Moreover, Tacrolimus reduced TLR5 expression in bladder macrophages during UTI. This immunosuppressive state can be explained by the upregulation of TLR-signaling negative regulators (A20, ATF3, IRAK-M and SOCS1) and parallel downregulation of TLR5 as observed in Tacrolimus treated granulocytes and macrophages. We conclude that Tacrolimus impairs host innate immune responses against UTI.

## Introduction

Urinary tract infection (UTI) is the most common bacterial infection in renal transplant patients^1^. The incidence of UTIs after kidney transplantation ranges from 6% to 86%^2,3^. UTIs leading to acute pyelonephritis can contribute to graft loss and mortality^4^. In the majority of cases, *E*. *coli* is the causative agent of UTIs^5^. The standard use of trimethoprim-sulfamethoxazole prophylaxis within the first 6 month after renal transplantation seems to be ineffective to prevent the high incidence and harm of UTIs^6^. One of the risk factors for developing UTI after renal transplantation is the lifelong use of immunosuppression in order to prevent acute graft rejection^7^. Calcineurin inhibitor Tacrolimus with its potent inhibitory effects on adaptive immunity, is one of the predominant anti-rejection agents used nowadays^8^.

A proper innate immune response is of great importance in clearing bacterial infections and mainly dependent on pathogen recognition by pattern recognition receptors (PRR) such as Toll-like receptors (TLRs)^9–11^. Previous studies have shown that TLR4, TLR5 and TLR11 play a crucial role in the immune reaction against UTIs^12–14^. Activation of the NF-κB pathway via TLRs in tissue resident leukocytes with invading *E*. *coli* leads to a robust inflammatory response^10^. Subsequently, released chemokines attract granulocytes from circulation to the infected tissues via the CXCR2 chemokine receptor^15^. Granulocytes control the infection by various mechanisms including the ability to produce oxidative burst and phagocytosis of pathogens^16^. This teamwork between tissue resident myeloid cells and circulating granulocytes is required to properly clear UTIs^9,17^.

Calcineurin is a negative regulator of the TLR-mediated activation pathway by interacting with MyD88, TRIF, TLR2 and TLR4^18^. It has been shown that calcineurin inhibition by Tacrolimus decreases responsiveness to LPS in macrophages and protects against LPS-induced toxicity in mice^19^. In human studies, it has been implicated that both calcineurin inhibitors Tacrolimus and Cyclosporine A have inhibitory effects on TLR signaling of myeloid cells post liver transplantation^20^. Moreover, a study showed that Cyclosporine A impairs Nucleotide-Binding Oligomerization Domain-Containing Protein 1 (NOD1)-mediated innate antibacterial renal defenses in mice and transplanted patients^21^. Despite the known interaction between calcineurin inhibitors and TLR pathway, it remains largely unknown how Tacrolimus may affect host antimicrobial defense mechanism against UTIs. Therefore, the aim of this study was to investigate if and how Tacrolimus suppresses the innate immune response against lower and upper UTI.

## Results

### Tacrolimus enhances the susceptibility to cystitis, pyelonephritis and bacteremia

**Figure 1:**
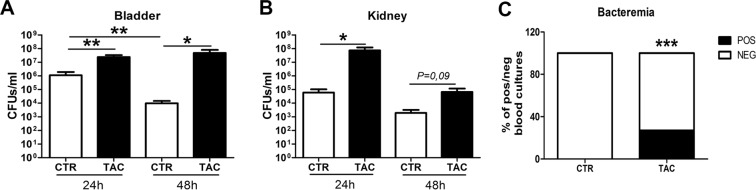
Higher bacterial load in Tacrolimus pre-treated mice during urinary tract infection. Bacterial load was quantified by determining colony forming units/ml in total bladder (**A**) and kidney (**B**) homogenates after 24 and 48 h of infection in solvent (CTR) and Tacrolimus (TAC) pre-treated mice. Percentage of bacteremia after 24 h (**C**). Data are expressed as mean ± SEM in (**A**,**B**). *P ≤ 0.05, **P < 0.01 and ***P < 0.0001. N = 8/group.

To investigate if Tacrolimus impairs host immune defense against lower and upper UTI, we induced UTI by intravesical inoculation with *E*. *coli* strain 1677 with solvent or Tacrolimus pre-treated mice and subsequently examined bacterial outgrowth in bladder and kidney homogenates 24 and 48 h later. At both time points, Tacrolimus pre-treated mice displayed higher amount of *E*. *coli* colony forming units (CFUs) in both organs (Fig. 1A,B). Blood cultures remained negative in all control mice, while 27% of Tacrolimus pre-treated mice showed positive blood cultures after 24 h of infection (Fig. 1C). These data demonstrate that Tacrolimus increases susceptibility to cystitis, pyelonephritis and bacteremia.

### Tacrolimus decreases primary functions of granulocytes

**Figure 2:**
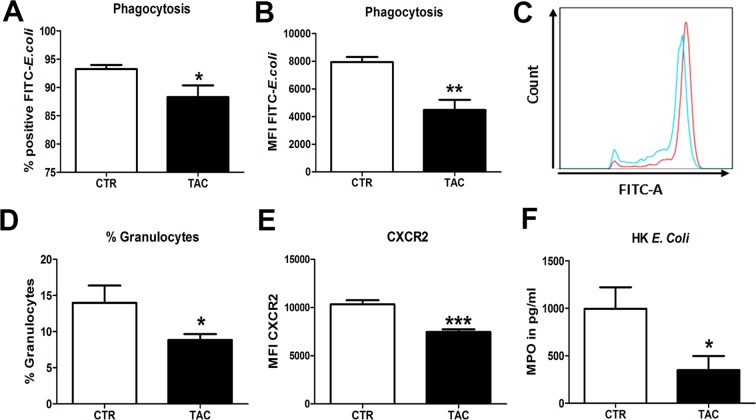
Tacrolimus suppresses primary functions of granulocytes. Percentage (**A**) and MFI (**B**) of FITC-labelled *E*. *coli* positive granulocytes from Control (CTR) and Tacrolimus (TAC) pre-treated mice. A representative FACS plot of FITC-positive granulocytes from Control (red) and Tacrolimus (blue) treated mice (**C**). Percentage of circulating granulocytes after 3 h of infection in Control and Tacrolimus pre-treated mice (**D**). MFI of CXCR2 on blood granulocytes after 3 h of infection in Control and Tacrolimus pre-treated mice (**E**). MPO release in response to HK *E*. *coli* by BM-granulocytes isolated from Control and Tacrolimus pre-treated mice (**F**). Data are expressed as mean ± SEM. *P ≤ 0.05, **P < 0.01 and ***P < 0.01. N = 5/8 per group.

Granulocytes are the first cells that are recruited to the bladder in the early hours of UTI thereby playing a major role in bacterial clearance^16^. Granulocytes control the infection via several mechanisms, including the upregulation of CXCR2 chemokine receptor, which enables their migration towards the infected tissue. Upon entry into urinary tract, granulocytes release MPO and phagocytose *E*. *coli*^15,16^. Granulocyte influx is closely related to bacterial burden during UTI, in other words; more CFUs leading to more granulocyte recruitment^15,16^. Since Tacrolimus pre-treatment resulted in higher bacterial burden, we wanted to test if Tacrolimus would affect the antimicrobial properties of granulocytes. We found that blood granulocytes from Tacrolimus pre-treated mice were less efficient in phagocytizing *E*. *coli ex vivo*, as reflected by the decreased percentage of *E*. *coli* positive granulocytes and lower MFI value compared to controls (Fig. 2A–C). Upon infection, percentage of circulating granulocytes and expression of CXCR2 on granulocytes were significantly reduced by Tacrolimus (Fig. 2D,E). BM-granulocytes from Tacrolimus pre-treated mice released moreover significantly less MPO in response to *E*. *coli* stimulation (Fig. 2F). Overall, granulocytes from Tacrolimus pre-treated mice have impaired antimicrobial properties.

### Tacrolimus decreases TLR5 expression in bladder macrophages during urinary tract infection

**Figure 3:**
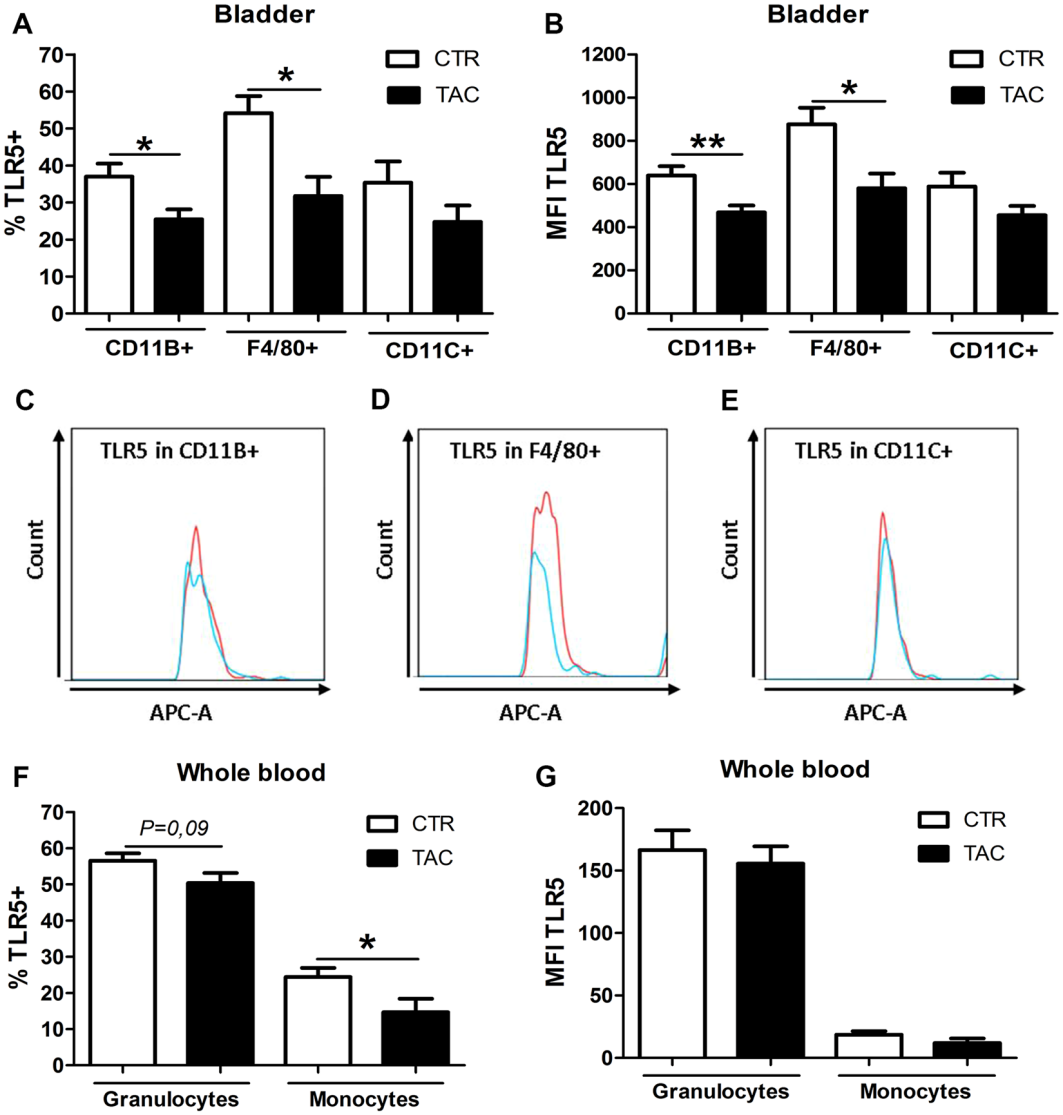
TLR5 expression is decreased in CD11b+ and F4/80+ bladder cells in Tacrolimus pre-treated mice during urinary tract infection. Percentage (**A**) and MFI (**B**) of TLR5 in CD11B+, F4/80+ and CD11C+ bladder populations with its FACS plots (**C**–**E**) after 20 h of infection in solvent (CTR) and Tacrolimus (TAC) pre-treated mice. Percentage (**F**) and MFI (**G**) of TLR5 in blood granulocytes and monocytes after 20 h of infection in Control (CTR) and Tacrolimus (TAC) pre-treated mice. Data are expressed as mean ± SEM. *P ≤ 0.05 and **P < 0.01. N = 8 per group.

Prior to granulocyte infiltration, invading *E*. *coli* is recognized by several TLRs including TLR4, TLR5 and TLR11^12–14^. TLR4 and TLR5 are expressed in kidney and bladder cells while TLR11 is only expressed in the kidney^10,12–14^. Gene expression analysis of these TLRs in kidney and bladder homogenates revealed that only TLR5 was reduced in the bladder of Tacrolimus pre-treated mice upon infection (Supplementary Fig. 1). To examine in which bladder cells TLR5 expression was reduced, we performed flow cytometry analysis of the bladder tissue. This revealed that the percentage of TLR5+ cells and the MFI of TLR5 was reduced in the CD11b+ and F4/80+ cells but not in the CD11c+ population (Fig. 3A–E) and non-immune cells (Supplementary Fig. 2). The percentage of TLR5+ monocytes in the circulation was moreover significantly lower in the Tacrolimus group, whereas in granulocytes there was only a decreased trend (*P* = *0*,*09*) in TLR5 positivity (Fig. 3F). The MFI of TLR5 in these cells remained steady (Fig. 3G). In conclusion, Tacrolimus reduces TLR5 expression mainly in the bladder macrophages during UTI.

### Tacrolimus interferes with TLR-pathway via inhibition of TLR-negative regulator calcineurin

**Figure 4:**
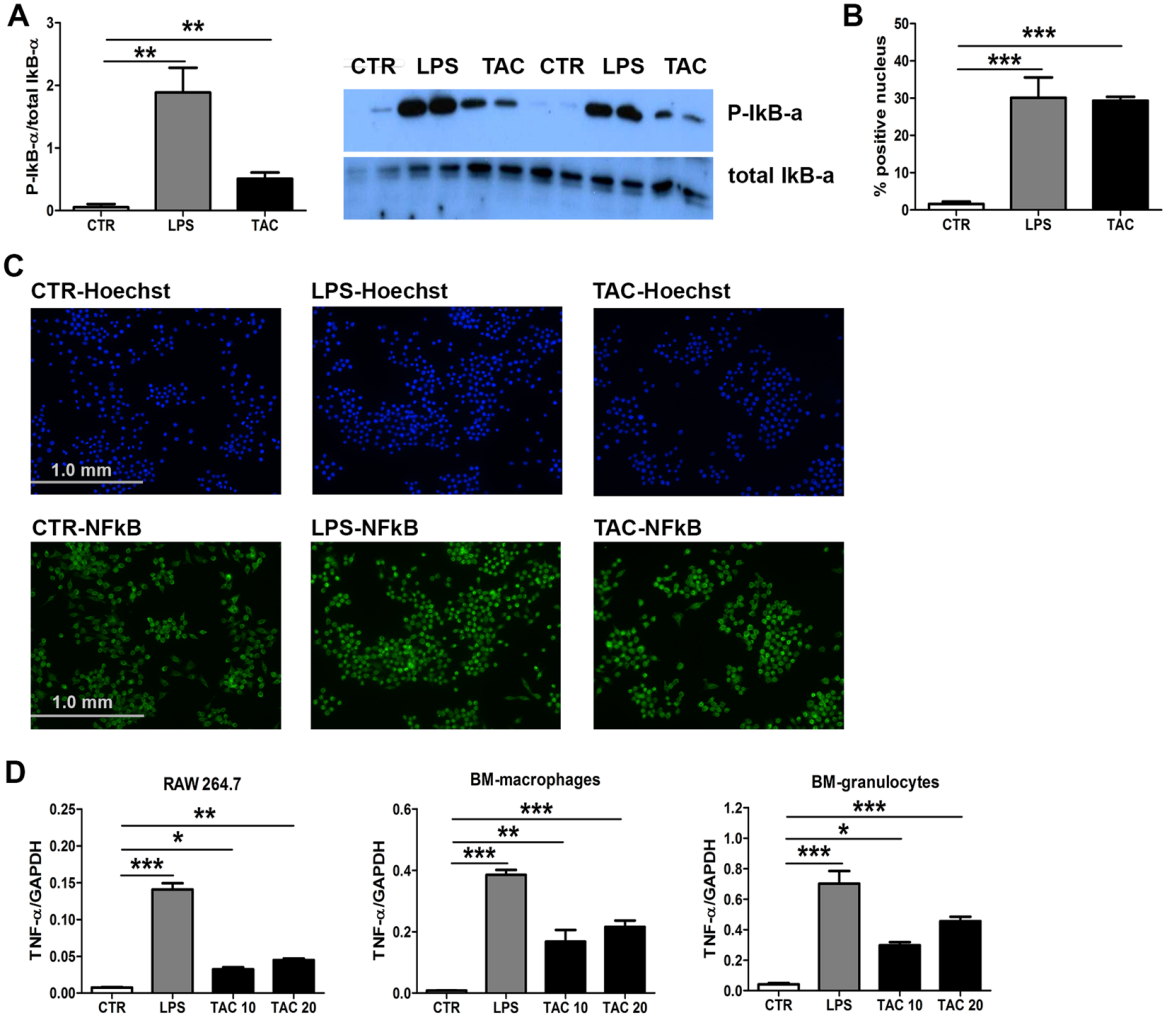
Tacrolimus induces activation of the classical NF-κB pathway. Protein expression of phospho-IκB-α corrected for total IκB-α (same gel) in RAW 264.7 macrophages stimulated with solvent (CTR), 100 µg/ml LPS or 20 µg/ml Tacrolimus (TAC) for 1 h (**A**), full-length blots are presented in Supplementary Fig. 3. Percentage of NF-κB positive nuclei (**B**). Representative stainings of nuclear translocation of NF-κB in RAW 264.7 macrophages stimulated with solvent, 100 µg/ml LPS or 20 µg/ml Tacrolimus for 1 h (**C**). mRNA expression of TNF-α in RAW 264.7 macrophages, BM-macrophages and BM-granulocytes after stimulation with either solvent, 100 µg/ml LPS or 10 and 20 µg/ml Tacrolimus for 4 h (**D**). Data are expressed as mean ± SEM. *P ≤ 0.05, **P < 0.01 and ***P < 0.01. N = 4 per group.

To understand how Tacrolimus induces an immunosuppressive state in myeloid cells, we next investigated the relation between calcineurin and TLR signaling. Calcineurin is a negative regulator of the TLR-mediated activation pathway by inhibiting the adaptor proteins MyD88 and TRIF^18^. Therefore, we treated macrophages either with solvent, LPS (positive control) or Tacrolimus and determined activation of TLR-mediated pathway. Both stimulations with LPS or Tacrolimus lead to phosphorylation of IκB-α (Fig. 4A) and nuclear translocation of the NF-κB into the nucleus as assessed by western blot and immunofluorescent staining, respectively (Fig. 4B,C). In line with these results, TNF-α mRNA was upregulated after LPS and Tacrolimus stimulation in macrophages and granulocytes (Fig. 4D). Thus, Tacrolimus alone activate NF-κB pathway and hence may influence TLR pathway in myeloid cells.

### Tacrolimus upregulates TLR negative regulators in macrophages and granulocytes

**Figure 5:**
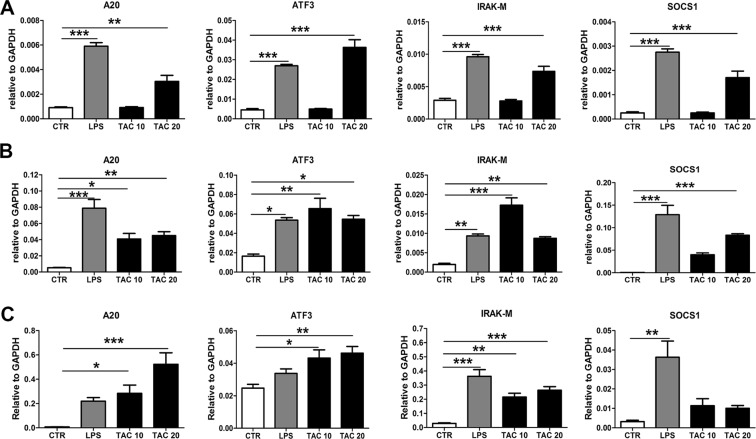
Tacrolimus induces upregulation of TLR negative regulators. mRNA expression of A20, ATF3, IRAK-M and SOCS1 in RAW 264.7 macrophages (**A**), BM-macrophages (**B**) and BM-granulocytes (**C**) after stimulation with either solvent (CTR), 100 µg/ml LPS or 10 and 20 µg/ml Tacrolimus (TAC) for 4 h. Data are expressed as mean ± SEM. *P ≤ 0.05, **P < 0.01 and ***P < 0.01. N = 4 per group.

LPS-induced NF-κB activation in myeloid cells eventually leads to endotoxin tolerance through upregulation of negative regulators of TLR-signaling that prevent excessive inflammatory reactions^22,23^. Since Tacrolimus activates NF-κB, we hypothesized that stimulation of myeloid cells with Tacrolimus might also result in the induction of negative regulators similarly to LPS stimulation. In line with our hypothesis, we found that the main NF-κB negative regulators; A20, ATF3, IRAK-M and SOCS1 were all increased in a macrophage cell line (Fig. 5A), Bone marrow (BM)-macrophages (Fig. 5B) and BM-granulocytes (Fig. 5C) after Tacrolimus stimulation as after LPS stimulation.

### Tacrolimus induces tolerance to LPS in macrophages

**Figure 6:**
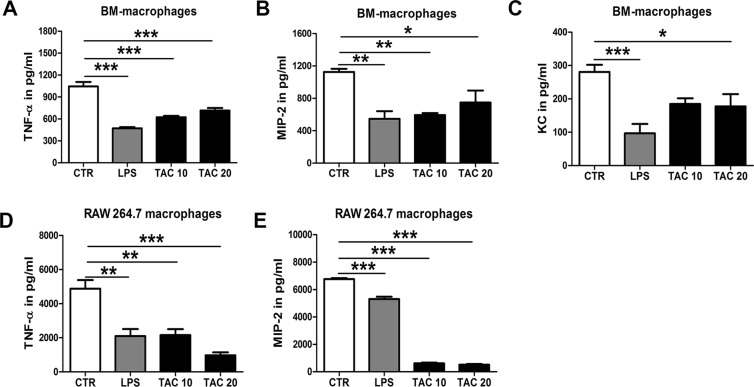
Tacrolimus induces a LPS-tolerant state. BM-macrophages (**A**) and RAW 264.7 macrophages (**B**) pre-treated with either solvent (CTR), 100 µg/ml LPS or 10 and 20 µg/ml Tacrolimus (TAC) for 24 h, after washing steps cells were re-stimulated with 100 ng/ml LPS for 4 h. Release of TNF-α, MIP-2 and KC was measurement in cell supernatants. Data are expressed as mean ± SEM. *P ≤ 0.05, **P < 0.01 and ***P < 0.01. N = 4 per group.

To verify whether the observed increased expression of negative regulators in Tacrolimus stimulated cells would lead to endotoxin tolerance, we pre-treated macrophages either with LPS (positive control) or Tacrolimus for 24 hours after which cells were washed and re-stimulated with a second “hit” of LPS. Indeed, Tacrolimus pre-treated macrophages were significantly less responsive to LPS as reflected by the remarkably reduction of TNF-α, MIP-2 and KC release (Fig. 6). Taken together, similar to endotoxin tolerance mechanism, Tacrolimus induces tolerance to LPS via induction of negative regulators in macrophages.

### Tacrolimus decreases TLR5 expression in granulocytes and macrophages

**Figure 7:**
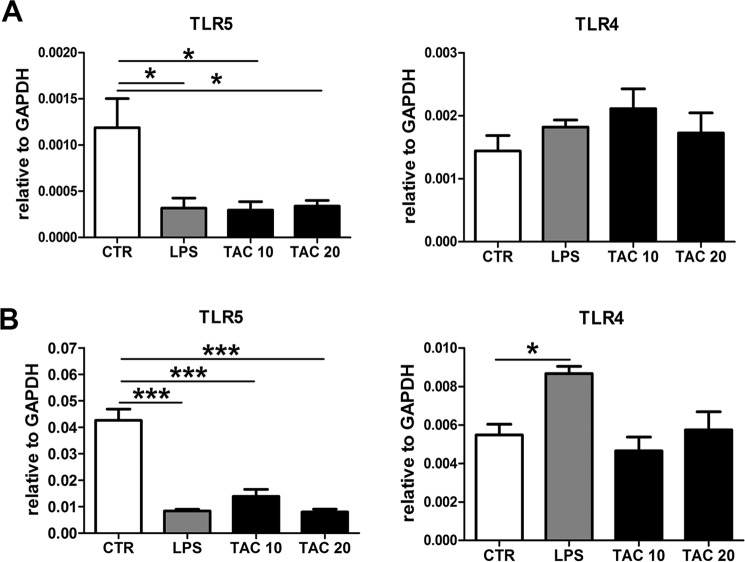
Tacrolimus reduces TLR5 expression during tolerant state. mRNA expression of TLR5 and TLR4 in BM-macrophages (**A**) and BM-granulocytes (**B**) after stimulation with either solvent (CTR), 100 µg/ml LPS or 10 and 20 µg/ml Tacrolimus (TAC) for 4 h. Data are expressed as mean ± SEM. *P ≤ 0.05, **P < 0.01 and ***P < 0.01. N = 4 per group.

Next to the induction of TLR negative regulators, downregulation of TLR expression could also be a mechanism by which cells prevent an uncontrolled inflammatory response^22^. As Tacrolimus reduced the expression of TLR5 in bladder macrophages during UTI, we analyzed the expression of TLR5 and TLR4 in BM-macrophages and –granulocytes. Strikingly, we found that similarly to LPS stimulation, Tacrolimus treatment strongly decreased TLR5 expression but not TLR4 in BM-macrophages and BM-granulocytes (Fig. 7A,B).

### Whole blood cells of renal transplant patients respond less to LPS and heat-killed *E*. *coli*

**Figure 8:**
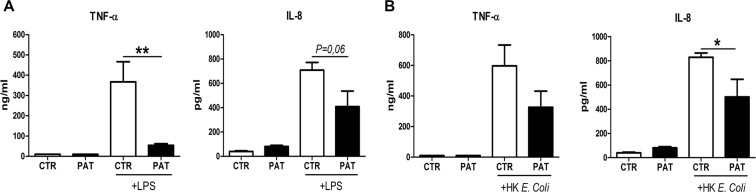
Leukocytes from renal transplant patients respond less to LPS and *E. coli* stimulus. Release of TNF-α and IL-8 after LPS (**A**) or *HK*. *E*. *Coli* stimulus by whole blood cells from healthy controls (CTR) and renal transplant patients (PAT). Data are expressed as mean ± SEM. *P ≤ 0.05 and **P < 0.01. N = 5 per group.

Next, we determined the antimicrobial properties of myeloid cells from renal transplant patients under Tacrolimus treatment as part of their immunosuppressive drug regimens. We did not find any differences in MPO release and phagocytosis capacity of granulocytes (data not shown), meaning that the main granulocyte functions are unaffected in these patients. However, in line with our *in vitro* and *in vivo* results in mice, we found that whole blood leukocytes from Tacrolimus-receiving patients release less TNF-α and IL-8 (human equivalent for murine KC) after LPS stimulation as compared to whole blood cells from healthy individuals (Fig. 8A). This trend was also seen after *E*. *coli* stimulation (Fig. 8B). These data indicate that myeloid cells from Tacrolimus-receiving individuals are less responsive to LPS/*E*. *coli* stimulation.

## Discussion

Given the high susceptibility of transplant patients to bacterial infections^1–3,7^, this study aims to shed light on the potential implication of Tacrolimus treatment to the higher incidence of UTI. Present study shows that Tacrolimus impairs immune responses against UTI by inhibiting several crucial parts of the antimicrobial defense mechanism. We found that mice pre-treated with Tacrolimus had higher bacterial burden (cystitis, pyelonephritis and bacteremia) upon UTI due to impaired granulocyte and macrophage response. Firstly, granulocytes from Tacrolimus pre-treated mice were less efficient in phagocytizing *E*. *coli;* secondly, they produced less MPO upon stimulation with *E*. *coli*; lastly, the chemokine receptor CXCR2 was markedly decreased in circulating granulocytes in the early hours of infections. Moreover, TLR5 which is crucial in defense against UTIs by recognizing bacterial flagellin^24^ was significantly reduced in bladder macrophages during infection. These effects appear to be driven by the induction of an immune-tolerant state by Tacrolimus. In macrophages, Tacrolimus pre-treatment lead to reduced responsiveness to LPS, as reflected by decreased release of TNF-α, KC and MIP-2. Furthermore, Tacrolimus activates the classical NF-κB pathway and induces the expression of its negative regulators A20, ATF3, IRAK-M and SOCS1 in both macrophages and granulocytes. This is likely due to the disruption of calcineurin-mediated inhibition of NF-κB signaling transduction by Tacrolimus as supported by the literature^18,19^. Taken together, our data show that the immunosuppressive state caused by Tacrolimus in macrophages and granulocytes results in higher bacterial burden during UTI.

A teamwork between granulocytes and macrophages is required for an optimal host defense against *E*. *coli* in the urinary tract system^17^. Resident macrophages in the bladder act like sentinels, recognizing *E*. *coli* via TLRs and responding to it by releasing pro-inflammatory mediators which in turn will attract granulocytes to the infected tissue. Although the role of macrophages during UTI is not extensively investigated as compared to granulocytes, two recent studies have highlighted the importance of macrophages during immune response against UTI^17,25^. Resident F4/80+ macrophages are the most populous immune cells in the naïve murine bladders^25^. Macrophages are the principal cells that acquire *E*. *coli* at early time points post infection and start to produce TNF-α, KC and MIP-2 in order to coordinating the exact recruitment and response of granulocytes in the infected urothelium^17,25^. Here, we show that Tacrolimus decreases macrophages response to LPS by dampening TNF-α, MIP-2 and KC production. In line with our results, another study has demonstrated that Tacrolimus inhibits macrophage inflammatory response, leading to impaired granulocyte recruitment and defective clearance of *Aspergillus fumigatus*^26^. In regard to granulocytes, our data show that the major functions of granulocytes, such as phagocytosis and MPO release are impaired by Tacrolimus. Accordingly, two independent studies showed that another calcineurin inhibitor Cyclosporine A impairs phagocytosis of granulocytes during UTI^21^ and that neutrophils lacking calcineurin are defective in killing *Candida albicans*^27^. We also found that the percentage of circulating granulocytes during UTI was reduced by Tacrolimus. In line, expression of the main receptor CXCR2, which is responsible for granulocyte recruitment during bacterial infections, was reduced on granulocytes by Tacrolimus during UTI. Indeed, CXCR2 receptor KO mice have delayed granulocyte transmigration across the urothelium leading to increased susceptibility to UTI^28^.

The antimicrobial immune response begins with the recognition of the pathogen by TLRs^10^. To date, TLR11, TLR4 and TLR5 have been described to play a role in the immune defense against UTIs *in vivo*^12–14,29^. TLR11 which is a pseudogene in humans, is mainly expressed by kidney cells and controls bacterial invasion of the kidney with no significant role in the bladder^14^. TLR4, instead, is expressed in both kidney and bladder^13^. TLR5 is essential in defense mechanism against UTIs and is found in both kidney and bladder^12^. The TLR11 and TLR4 expression during UTI was unchanged by Tacrolimus, whereas expression of TLR5 was specifically reduced in bladder macrophages. Feuillet *et al*. showed that the initial inflammatory response in the bladder is TLR5-dependent^30^. In support of these findings, TLR5 polymorphism in adult women is associated with increased risk to UTIs but not pyelonephritis^31^, most presumably because TLR5 does not play a role in the kidney.

TLR-activation must be tightly regulated to prevent an exacerbated inflammatory response. The main mechanisms by which myeloid cells regulate TLR-mediated signaling is either via reducing TLR expression or via induction of negative regulators^22,23^. As Tacrolimus inhibits calcineurin, which in turn is an inhibitor of the MyD88 and TRIF-pathways^18^. We hypothesized that inhibition of a TLR-pathway inhibitor would lead to activation of NF-κB and, consequently, to induction of its negative regulators in myeloid cells. We found that several NF-κB negative regulators (IRAK-M, A20, ATF3 and SOCS1) were upregulated after Tacrolimus stimulation in macrophages and granulocytes, thereby triggering a tolerant state in which myeloid cells become less responsive to LPS or *E*. *coli*. Indeed, Tacrolimus decreased the secretion of TNF-α, MIP-2 and KC by macrophages in response to a secondary LPS stimulation and attenuated granulocytes responsiveness to *E*. *coli*. Nonetheless, the LPS receptor TLR4 expression was unchanged by Tacrolimus and also during endotoxin tolerance^32^. Although the role of TLR5 during endotoxin tolerance is still unknown, our data show that TLR5 expression is significantly decreased in macrophages and granulocytes activated by Tacrolimus. Since this also happens after LPS stimulation and contemporary to the upregulation of NF-κB negative regulators, it might be argued that the downregulation of TLR5 is a characteristic of endotoxin tolerance. To summarize, our data show that Tacrolimus, similarly to endotoxin tolerance-phenomena, induces tolerance in myeloid cells. This immunosuppressive state occurs by fading the calcineurin-mediated NF-κB signaling inhibition and subsequently by inducing the NF-κB-dependent induction of its negative regulators which in turn reduce further response to endotoxin. Endotoxin tolerance is related to impaired resistance against several infectious complications^33,34^. Presumably, the immunotolerant state caused by Tacrolimus inhibits the immune response against UTI in the same manner.

It must be mentioned that concentrations of Tacrolimus used in our experimental setup should not be compared to concentrations achieved in patients. Drug pharmacokinetics and pharmacodynamics differ a lot among species and therefore this difference should be taken under consideration during designing of an experiment^35^. For instance, mice can handle much higher drug concentrations compared to humans. The concentration of 5 mg/kg/day *in vivo* is a widely used dose of Tacrolimus in mouse models^26,36^. This particular dose has even been shown to be most effective in nerve transplantation in mice^36^. Nevertheless, during the whole *in vivo* experiment, mice were critically observed for signs of discomfort, by checking the body weight and behavior. Weight loss in mice is a strong indicator for toxicity during drug treatment. In our study, after 3 days of Tacrolimus treatment, mice did not lose weight compared to solvent-treated mice as shown in Supplementary Fig. 4, which indicates that Tacrolimus dose was not toxic. In addition, these mice were behaving normal (no signs of decreased mobility).

Papers indicating an increased incidence of UTIs in renal transplant patients do not directly support a role of immunosuppression, not because they did not find an association but because they did not look at immunosuppressive regimen as a varying variable. Early UTIs (<6 month post transplantation) can indeed be explained in many cases by surgical procedure and anatomical position of the donor kidney. However, late UTIs are also common and not benign and this is most likely explained by the long-term use of immunosuppression, including Tacrolimus^4^. Therefore, all patients included in our study were >6 month post-transplantation. To support our hypotheses, high incidences of UTI in non-renal transplant patients is not uncommon (liver, heart and lung transplantation) with *E*. *coli* being the most common causative pathogen^37^, which indicates that non-anatomical, hence immunosuppression is related to high incidence of UTIs as well^38^. It has been revealed that in patients, Cyclosporine A and Tacrolimus regimes have inhibitory effects on TLR signaling post liver transplantations^20^. PBMCs from patients using Tacrolimus respond significantly less to TLR4-mediated TNF-α release^20^. Comparably, we found that leukocytes from whole blood of renal transplant patients receiving Tacrolimus have diminished TLR4-induced TNF-α release. However, we did not find any changes in the phagocytosis capacity and MPO release in granulocytes from the same patients. A previous study has shown that phagocytosis of *E*. *coli* was reduced in renal transplant patients, however these patients were being administered Cyclosporine A and not Tacrolimus^21^. It must be stressed that investigating innate immune cells from renal transplant patients is challenging because their immunosuppressive drug regimens do not include solely Tacrolimus/Cyclosporine A but a whole panel of other immunosuppressive agents as well. Renal transplant patients on only one immunosuppressive drug is very rare and therefore almost impossible to include in a study. Therefore, we have performed *in vivo* experiments with mice pre-treated either with MFF or Prednisone only, and found that the outcome of UTI was similar between CTR and drug treated mice (data not shown). This indicates that not MFF or Prednisone but Tacrolimus might be related to higher bacterial burden during UTI in mice.

The present study is the first one showing the dramatic effects of Tacrolimus on functionality of macrophages and granulocytes leading to impaired antimicrobial defense against UTI. The knowledge from this study can be used in the future to develop immunosuppressive agents which aim to selectivity inhibit the adaptive immunity, thereby preventing graft loss, with no negative effects on the innate immunity. In this way, renal transplant patients will gain a higher quality of life in which bacterial infections will no longer be a concern.

## Materials and Methods

### Mice

C57Bl/6 wildtype mice were purchased from Charles River (Maastricht, The Netherlands). Mice were housed under specific pathogen-free conditions and received water and food ad libitum. All animal experiments were approved by the Animal care and Use Committee of the University of Amsterdam, confirming that all experiments were performed in accordance with relevant guidelines and regulations.

### Tacrolimus treatment

8–12 weeks old female mice were pre-treated daily with an i.p. injection of 5 mg/kg Tacrolimus (LC laboratories) or solvent (olive oil) for 3 consecutive days. For *in vitro* experiments cells were incubated with 10 or 20 µg/ml of Tacrolimus, a concentration range that has been used previously^19,39,40^. Viability of cells treated with Tacrolimus was determined with MTT assay (Sigma Aldrich) (Supplementary Fig. 5). Tacrolimus was tested for endotoxin contamination with Toxin Sensor Gel Clot Endotoxin assay kit (GenScript) according to manufacturer’s instructions.

### Urinary tract infection

UTI was induced as described previously with small modification of the protocol^41–43^. Briefly, *E*. *coli* strain 1677, isolated from an uroseptic patient was grown overnight at 37 °C in Tryptone Soy Broth (TSB) medium, followed by 1:100 dilution in TSB medium. After reaching an optical density of 1 (OD_600nm_), *E*. *coli* was washed twice in ice-cold PBS and resuspended in 5 ml of PBS. Infection was induced by transurethral inoculation of 50 µl of *E*. *coli* suspension into the bladder by 0.55 µm catheter (Abbott). UTI was induced under general anesthesia, induction rate was 3% of Isoflurane and 1–2% for maintenance. After inoculation of *E*. *coli* into the bladder, mice were kept under anesthesia for 1 hour. Subsequently, under general anesthesia, mice were sacrificed after 3, 24 and 48 hours of infection by heart puncture followed by cervical dislocation.

### Bacterial outgrowth

Bacterial outgrowth was determined as described previously^41^. Briefly, bladder and the left kidney were homogenized in PBS with a tissue homogenizer. The homogenizer was cleaned twice with 70% alcohol and once with PBS after each sample. Tissue homogenates were diluted in serial 10-fold dilutions in PBS, 50 µl of homogenates and blood were plated out onto blood agar plates at 37 °C o/n. The next day, *E*. *coli* colony forming units (CFUs) were counted.

### ELISA

Cytokine, chemokine and MPO levels were measured in cell supernatants or bladder and kidney homogenates with specific ELISA DuoSet kits (R&D Systems) according to manufacturer’s instructions. The total amount of protein in tissue homogenates was measured with BCA assay.

### BM-granulocytes, BM-macrophages and RAW 264.7 macrophages

BM-granulocytes and BM-macrophages were isolated/generated as described previously^44^. Briefly, femoral and tibial bone marrow was flushed with ice-cold sterile PBS with a 21-gauge needle above a 40 µm pore filter. Granulocytes were isolated by means of magnetic labeling using anti-Ly6G Microbead Kit, according to manufacturer’s instructions (Miltenyi Biotec). BM-macrophages were generated by culturing total bone marrow cells in petri dishes (Greiner) with bone marrow macrophage medium (RPMI culture medium (Gibco) supplemented with 10% FCS, 100IU/ml penicillin, 100 µg/ml streptomycin, 2mM L-Glutamine (Invitrogen) and 15% of LCM (L929 conditioned medium)) for 7 days. RAW 264.7 macrophage cell line was cultured in DMEM (Gibco) culture medium containing 10% FCS, 100 IU/ml penicillin, 100 µg/ml streptomycin, 2 mM L-Glutamine (Invitrogen). Cells were passaged by ~80% confluency. Macrophages were stimulated with solvent, 100 ng/ml LPS, 10 or 20ug/ml of Tacrolimus for 24 hours, after which cells were washed 3 times with PBS and re-stimulated with 100 ng/ml LPS for 4 hours. Supernatants were collected and stored at −20 °C until analysis. To determine expression of negative regulators on mRNA level, cells were stimulated with the same concentrations of LPS or Tacrolimus for 4 hours, followed by treatment with Tri-reagent after which samples were collected and stored at −80 °C for further process.

### Reverse transcriptase-PCR

Kidney, bladder homogenates and cells were treated with Tri-reagent (Sigma Aldrich) to isolate total RNA according to the manufacturer’s protocol. cDNA was synthesized using oligo-dt as primer. mRNA expression of several genes was measured by RT-PCR performed on a Light Cycler 480 (Roche) with SYBR green PCR master mix (Bioline). Intensity of SYBR green dye was determined by linear regression analysis (LinRegPCR, developed by Heart Failure Research Center, Amsterdam, The Netherlands). Expression of specific genes were normalized to expression of the house keeping gene GAPDH.

### Phagocytosis assay

Phagocytosis of *E*. *coli* by blood granulocytes was measured with PHAGOTEST (Glycotope Biotechnology) according to the manufacturer’s instructions. Briefly, 100 µl of heparinized whole blood was stained with opsonized FITC-labelled E.coli and incubated for 10 minutes at 37 °C (water bath), while the negative control remained on ice. After phagocytosis was stopped, quenching solution was used to discriminate between surface bound and internalized *E*. *coli*. After lysing of the red blood cells and DNA staining to discriminate *E*. *coli* from granulocytes, phagocytic capacity was measured by flow cytometry on a FACS Canto (BD Bioscience) and analyzed with FlowJo version 10.

### Western blotting

Macrophages were lysed in RIPA buffer supplemented with 4 mM Na_3_VO_4_, 10 mM NaF and 1:100 protease inhibitor (Sigma Aldrich). Protein concentrations were determined by BCA assay. Cell lysates were denatured with 2-mercaptoethanol and incubated at 95 °C for 8 min, 20ug protein was loaded on a 12% SDS-PAGE gel and blotted on a blot membrane (Millipore). The blot was cut in two before incubation with the antibodies. Blots for phospho- IκBα were incubated in blocking buffer (Tris buffered saline supplemented with 0.1% Tween 20 (Sigma Aldrich)) containing 5% milk or for total IκBα in blocking buffer with 5% BSA for 1 hour). Blots were incubated with primary mouse anti-phospho-IκBα (Cell Signaling) 1:1000 antibody in blocking buffer o/n. As secondary antibody, goat anti-mouse IgG1-hrp (1:1000 in blocking buffer) (Sigma Aldrich) was used. For total IκBα, rabbit anti-mouse polyclonal IκBα (1:500) (Santa Cruz) and as secondary antibody goat anti-rabbit-hrp (1:1000) (Sigma Aldrich) was used. Samples were detected with ECL+ (Amersham) using autoradiography films (GE Healthcare). Western blot band were quantified with Image J software.

### Immunofluorescence staining

Macrophages were plated out at a concentration of 0.05*10^6^ cells/well on coverslips in a 24-wells plate (Greiner). After 2 days, cells were stimulated with solvent, 100 ng/ml LPS or 20 µg/ml Tacrolimus for 1 hour. Cells were fixed with 4% formaldehyde for 10 minutes, followed by permeabilization and blocking step with TBP (0.1% Triton X-100, 0.5% BSA in PBS) for 30 minutes. Samples were incubated with primary antibody rabbit anti-mouse NFκB (Santa Cruz) 1:500 in TBP o/n at 4 °C. After washing step, cells were incubated with the secondary antibody goat anti-rabbit-Alexa Fluor 488 (Life Technologies) at a dilution of 1:1000 in TBP for 1 hour at RT. The nuclei were stained with 0.5 µg/ml Hoechst in PBS for 30 minutes at RT. Pictures were made with Leica epifluorescence microscope software. Translocation of NFκB was determined by counting total and NF-κB positive nuclei in a blinded fashion.

### White blood cells

Composition of whole blood was determined with Scil Vet abc Plus+ (HORIBA Medical) in 30 µl blood sample.

### Flow cytometry

Bladder tissue was minced and digested with digestion buffer containing 0,34 U/ml Liberase TM and 100 µg/ml DNase (Sigma Aldrich) in PBS for 1 hour at 37 °C. Cells were washed with FACS buffer and passed through a 100 µm cell strainer, blocked with anti-Fc Receptor (Anti-Mouse CD16/CD32, eBioscience) and stained with antibodies anti-CD45.2-Percp Cy5.5, CD11b-FITC, F4/80-PE or CD11C-PE and TLR5-APC (Biolegend) in FACS buffer. Stainings were visualized using a BD LSRFortessa™ cell analyser (BD Bioscience); DAPI (Sigma-Aldrich) was added to the cell suspension short before measurement to discriminate dead from alive cells. Flow cytometry data were analyzed using FlowJo v10 (Ashland, OR).

### Renal transplant patients

Patient selection criteria were as follow: men and women >18 years old, at least 6 month after renal transplantation, on Tacrolimus, MMF and prednisolone >3 month, clinically stable at the time of blood donation. Healthy controls were matched with age and gender of the patients. Heparinized blood was collected and *ex vivo* stimulated with 500 ng/ml LPS or HK *E*. *coli* for 4 h hours, after which the supernatants were stored at 20 °C for analysis of cytokines. All human subjects signed an informed consent. This study was approved by the Medical ethical committee of the AMC, confirming that all experiments were performed in accordance with relevant guidelines and regulations.

### Statistical analyses

All statistical analyses were performed using Graphpad Prism version 5 software. Results are expressed as mean ± SEM. Comparisons between two groups were analysed using the two-tailed unpaired *t* test. One-way ANOVA followed by Bonferroni’s multiple comparison test was used for comparison between more than 2 groups. Fisher’s exact test was used for analysis of bacteremia. Values of *P* ≤ 0.05 were considered to represent a statistically significant difference.

## Supplementary information

supplementary info
