# Supplementary material for: Calcineurin inhibitor Tacrolimus impairs host immune response against urinary tract infection
Source: Sci Rep. 2019 Jan 14;9:106. doi: 10.1038/s41598-018-37482-x (PMC6331640; doi:10.1038/s41598-018-37482-x)
Supplement: Supplementary file 1 — supplementary info [file 41598_2018_37482_MOESM1_ESM.pdf]

Supplementary information for the following manuscript:

Title: Calcineurin inhibitor Tacrolimus impairs host immune response against urinary tract infection

Authors: Diba Emal, Elena Rampanelli, Nike Claessen, Frederike J. Bemelman, Jaklien C. Leemans, Sandrine Florquin and Mark C. Dessing

Supplementary figures

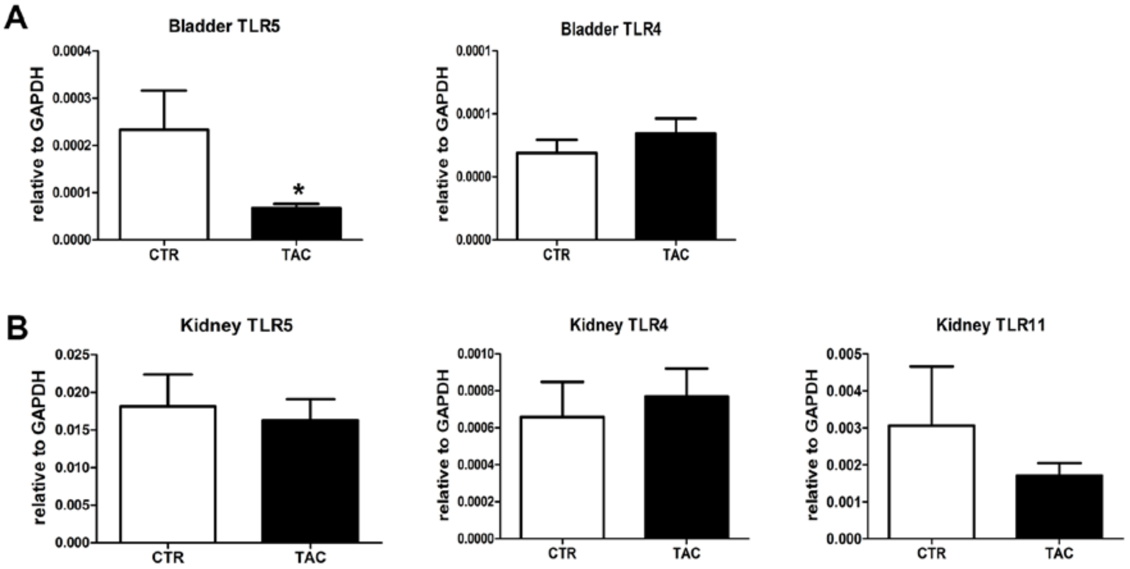

Supplementary figure 1: **Tacrolimus reduces TLR5 expression in bladder tissue during UTI**. mRNA expression of several TLRs in bladder (A) and kidney (B) tissue during UTI in solvent (CTR) and Tacrolimus (TAC) pre-treated mice. Data are expressed as mean ± SEM. N=8 per group.

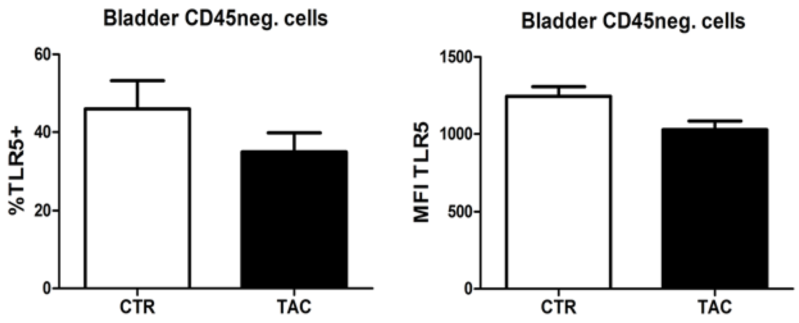

Supplementary figure 2: Expression of TLR5 in bladder non-immune cells during UTI. Percentage and MFI of TLR5 in CD45 negative bladder cell population during infection in solvent (CTR) and Tacrolimus (TAC) pre-treated mice. Data are expressed as mean ± SEM. N=8 per group.

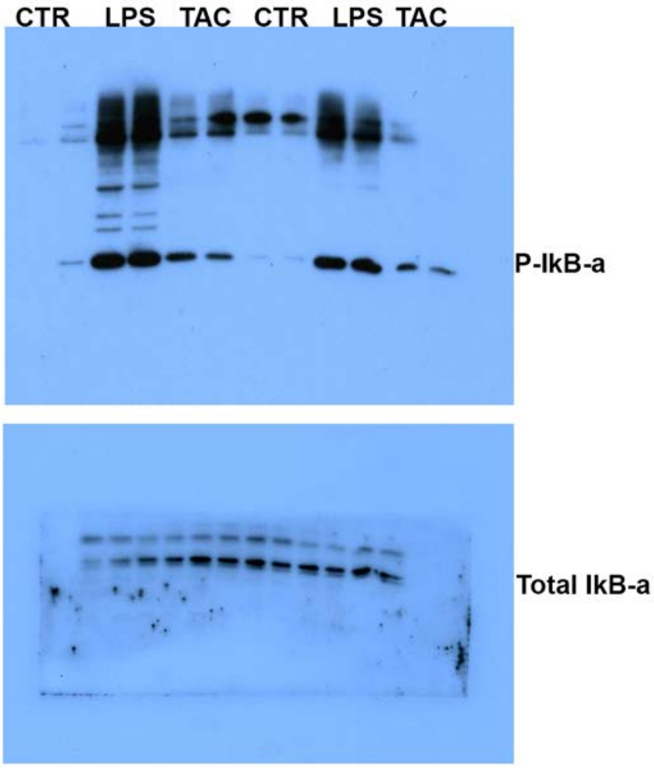

Supplementary figure 3: Full-length blots for protein expression of Total and p-IkB-α on the same gel. Blot was cut in two before incubation with specific antibodies. N=4 per group.

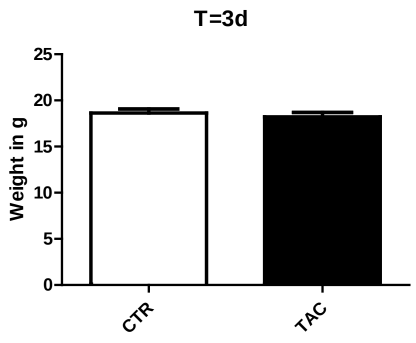

Supplementary figure 4: Weight of mice after 3 days of treatment either with solvent or Tacrolimus in grams. Data are expressed as mean ± SEM. N=8 per group.

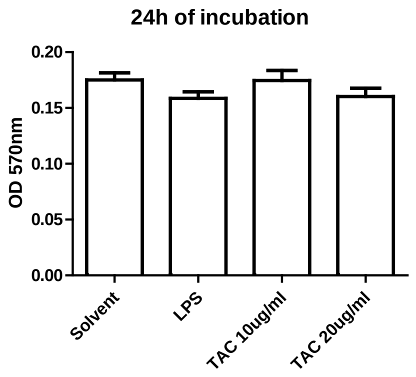

Supplementary figure 5: Viability of macrophages after 24 hours of incubation with Solvent, LPS or TAC 10-20ug/ml is comparable, as determined by MTT assay. Data are expressed as mean ± SEM. N=8 per group.
